# A conceptual framework for the study of demonstrative reference

**DOI:** 10.3758/s13423-020-01822-8

**Published:** 2020-10-09

**Authors:** David Peeters, Emiel Krahmer, Alfons Maes

**Affiliations:** Department of Communication and Cognition, TiCC, Tilburg University, P.O. Box 90153, NL-5000 LE Tilburg, The Netherlands

**Keywords:** Referential communication, Demonstratives, Pointing, Multimodal communication

## Abstract

Language allows us to efficiently communicate about the things in the world around us. Seemingly simple words like *this* and *that* are a cornerstone of our capability to refer, as they contribute to guiding the attention of our addressee to the specific entity we are talking about. Such demonstratives are acquired early in life, ubiquitous in everyday talk, often closely tied to our gestural communicative abilities, and present in all spoken languages of the world. Based on a review of recent experimental work, here we introduce a new conceptual framework of demonstrative reference. In the context of this framework, we argue that several physical, psychological, and referent-intrinsic factors dynamically interact to influence whether a speaker will use one demonstrative form (e.g., *this*) or another (e.g., *that*) in a given setting. However, the relative influence of these factors themselves is argued to be a function of the cultural language setting at hand, the theory-of-mind capacities of the speaker, and the affordances of the specific context in which the speech event takes place. It is demonstrated that the framework has the potential to reconcile findings in the literature that previously seemed irreconcilable. We show that the framework may to a large extent generalize to instances of endophoric reference (e.g., anaphora) and speculate that it may also describe the specific form and kinematics a speaker’s pointing gesture takes. Testable predictions and novel research questions derived from the framework are presented and discussed.

## Introduction: Demonstrative reference as a joint action

Although the capacity to communicate about entities beyond the here-and-now is a powerful design feature of human language (Hockett, 1960), we nevertheless also often talk about the things in our immediate surroundings. In everyday conversations, speakers indeed naturally exploit the communicative potential of words, gestures, and facial expressions to share their thoughts about people, objects, and ongoing events in their direct environment. It has long been acknowledged that referring to something in such face-to-face situations is a social and collaborative enterprise (Bara, 2010; H. H. Clark & Bangerter, 2004; H. H. Clark & Wilkes-Gibbs, 1986; Grice, 1975). When selecting from a wide range of possible referring expressions (cf. ‘that blue bicycle right there’ to ‘the bike’ to ‘it’), speakers typically take into account the presumed cognitive status of a referent in their addressee’s situation model (e.g., Ariel, 1988; Arnold, 2010; Chafe, 1976; Gundel, Hedberg, & Zacharski, 1993; Hanks, 2011; Prince, 1981b). Addressees, in turn, single out one or more referents based on the verbal and nonverbal information provided by the speaker considering their assumed common ground (H. H. Clark, 1996; H. H. Clark, Schreuder, & Buttrick, 1983).

The collaborative nature of referring in face-to-face communication is also evident from its multimodal characteristics. When physically pointing at a visible referent—for instance, by using the index finger—speakers typically alternate gaze between referent and addressee (Bakeman & Adamson, 1984; Kita, 2003) and tailor the kinematic properties of their gesture (Cleret de Langavant et al., 2011; Liu, Bögels, Bird, Medendorp, & Toni, 2019; Peeters, Chu, et al., 2015) and the specificity of concurrently produced speech (Brennan & Clark, 1996; H. H. Clark & Wilkes-Gibbs, 1986; Koolen, Gatt, Goudbeek, & Krahmer, 2011) to the presumed informational needs of their addressee. Addressees may use the vector created by the speaker’s gesture, available gaze cues, and any concomitant verbal description to establish joint attention to the inferred, intended referent (Bangerter, 2004; H. H. Clark, 2020; Cooperrider, 2020; Diessel, 2006; Eco, 1976; Kita, 2003; Levinson, 2004), subsequently verbally and nonverbally signaling their understanding to the speaker (H. H. Clark & Krych, 2004). As such, referring can be considered both a social and a multimodal hallmark of human communication (Peeters & Özyürek, 2016).

The current paper focuses on the production of demonstratives—deictic words like *this*, *that*, *these*, and *those—*as a central component of many such multimodal joint actions. As far as we know, all spoken languages have an inventory of these linguistic expressions (Diessel, 1999; Dixon, 2003), present in the lexicon of a language as a closed-class set of words or morphemes such as affixes or clitics (Diessel, 1999; Levinson, 2018). Demonstratives are among the earliest words infants produce (Capirci, Iverson, Pizzuto, & Volterra, 1996; E. V. Clark, 1978; E. V. Clark & Sengul, 1978), and their usage remains ubiquitous in face-to-face communication throughout life (Wu, 2004) as they occur in various common speech acts, for instance when we express our attitudes about something (‘that is a pretty flower’), provide our interlocutor with new information (‘this is your new colleague’), or point at something as a request or imperative for assistance (‘could you pass me that burrito, please?’). Frequency counts in lexical databases (e.g., Celex, Lexique, Subtlex) for various languages indeed consistently rank demonstratives amongst the most highly used lexical items in language (Baayen, Piepenbrock, & van Rijn, 1993; Brysbaert & New, 2009; Keuleers, Brysbaert, & New, 2010; New, Pallier, Brysbaert, & Ferrand, 2004). Historically, demonstratives are so old that they cannot easily be traced back to diachronically earlier linguistic expressions (Diessel, 1999; Himmelmann, 1996), suggesting that they might even be “the most basic communicative acts in the vocal modality” (Tomasello, 2008, p. 233). Not surprisingly, therefore, they have long been a topic of extensive study in various scientific disciplines such as philosophy (e.g., Kaplan, 1979; Peirce, 1940), psychology (Bühler, 1934; Kemmerer, 1999), cross-linguistic typology (e.g., Anderson & Keenan, 1985; Fillmore, 1982), linguistic anthropology (e.g., Enfield, 2003; Hanks, 1990), discourse studies (e.g., Ariel, 1990; Gundel et al., 1993), and foreign language learning (e.g., Petch-Tyson, 2000; Zhang, 2015). Furthermore, they play an important part in some of the most iconic works of art, from Magritte’s *ceci* *n’est pas une pipe* to Shakespeare’s *to be or not to be /* *that* *is the question*.

Despite the universal existence of demonstratives in all spoken languages (Diessel, 1999), the number of available demonstratives per language is a matter of remarkable cross-linguistic diversity (Diessel, 2013; Levinson, 2018; Weissenborn & Klein, 1982). Whereas English, for instance, distinguishes between a ‘proximal’ (*this* or *here*) and a ‘distal’ (*that* or *there*) form, it is not uncommon for languages to have three (e.g., Spanish, Japanese), four (e.g., Quileute, Somali), or even five or more (e.g., Malagasy, Navajo) different basic demonstrative terms (Diessel, 2013). Speakers of other languages (e.g., Modern French, German) may have only a single basic demonstrative determiner at their disposal, but can use a richer set of demonstrative adverbs similar to English’ *here* and *there* (Diessel, 2013; McCool, 1993). The existence of more than one demonstrative in a given language and the fact that languages cross-linguistically differ in the number of available terms naturally raises the question what factors drive a speaker in their decision to use one demonstrative form and not another in a specific context. Regardless of what exact factors may influence this selection process, it is within the larger framework of referring as a collaborative joint action (Bangerter, 2004; H. H. Clark, 1996) that a speaker’s implicit decision to use one demonstrative form (e.g., *this*) over another (e.g., *that*) should be situated (Enfield, 2003; Hanks, 2011; Jarbou, 2010; Peeters & Özyürek, 2016).

Complementing earlier philosophical, linguistic, and anthropological work that was predominantly based on ‘armchair intuitions’ and field observations (H. H. Clark & Bangerter, 2004), recent years have seen an increase in experimental research into the use and processing of demonstratives (e.g., Bonfiglioli, Finocchiaro, Gesierich, Rositani, & Vescovi, 2009; Coventry, Valdés, Castillo, & Guijarro-Fuentes, 2008; Peeters, Hagoort, et al., 2015; Rocca, Wallentin, Vesper, & Tylén, 2019). An important aim of many such well-controlled studies has indeed been to pinpoint precisely, often in carefully monitored lab settings, what factors (e.g., the location of a referent or its visibility) affect whether a speaker selects one demonstrative form and not another, and as such, what demonstratives implicitly tell the addressee about the relative location and/or cognitive status of the referent. This strictly experimental work from the lab is further complemented by quasi-experimental work performed at field sites around the world (e.g., Levinson et al., 2018; see also Da Milano, 2007) and in the lab (Maes & de Rooij, 2007; Piwek, Beun, & Cremers, 2008), and by work looking at why speakers use a demonstrative (versus an alternative referring expression) to start with (e.g., Bangerter, 2004; Cooperrider, 2016). Although the recent experimental approach to the study of demonstrative reference has yielded several interesting insights, we do not yet understand the mechanisms at work in the mind of a speaker when they select a demonstrative form for inclusion in their referential utterance. Moreover, a comprehensive account integrating the variety of observational and experimental findings at a cognitive level is lacking.

The main aim of the current paper is therefore, based on a review of the experimental literature on demonstratives situated in the broader context of earlier nonexperimental work, to introduce a conceptual framework that describes the various factors and mechanisms involved in demonstrative reference across languages. We will initially focus on situations in which speakers use demonstratives exophorically (i.e., in reference to entities present in the immediate surroundings of the speech event; Halliday & Hasan, 1976; Levinson, 1983) and show how the framework may explain a speaker’s choice of demonstrative form in different contexts. We will then explore whether the framework conceptually generalizes to cases of *endophoric* demonstrative reference (Levinson, 1983; Lyons, 1977), particularly situations in which speakers or writers refer anaphorically to elements of the ongoing discourse. We hope that the framework will serve as a conceptual basis for future experimental and observational work on demonstratives. Before introducing the framework, we will now first provide a review of recent experimental findings on demonstrative use across different languages.

## The experimental study of demonstratives: A review of recent work

The traditional view on demonstratives in exophoric use is that they “indicate the relative distance of a referent in the speech situation vis-à-vis . . . the speaker’s location at the time of the utterance” (Diessel, 2013, p. 1). In a nutshell, this speaker-centric spatialist account proposes that ‘proximal’ demonstratives (e.g., English *this*) are used in reference to entities relatively nearby the speaker, and ‘distal’ demonstratives (e.g., English *that*) in reference to entities relatively far from the speaker (Anderson & Keenan, 1985; Halliday & Hasan, 1976; Levelt, 1989). This “folk-view on proximal and distal demonstratives” (Piwek et al., 2008, p. 695) has been found to be too simplistic (e.g., Enfield, 2003; Hanks, 2009; Jarbou, 2010; Kemmerer, 1999; Peeters & Özyürek, 2016; Strauss, 2002), and extensive cross-linguistic experimental and observational work questions “whether any language actually has a system like this” (Levinson, 2018, p. 6). Based on a review of the experimental literature on demonstratives, we here suggest that rather at least three *types of factors* influence a speaker’s choice for a specific demonstrative form in any given setting. These three types of factors (physical, psychological, and referent-intrinsic) are proposed to play a role, to a variable extent, in all communicative situations in which a speaker uses a demonstrative in reference to an entity in the world.

### Physical factors influencing a speaker’s choice of demonstrative form

The experimental literature firstly suggests that *physical* factors play a role in influencing a speaker’s choice of demonstrative form. We here define physical factors as aspects of the external physical context in which language is used that can be objectively observed and determined, such as the relative physical distance of a referent in relation to the speaker or the speech situation, and a referent’s visibility to the interlocutors. Various instantiations of the *relative location* of a referent have indeed been proposed to influence a speaker’s decision to use one specific demonstrative form over another. A series of experiments has made clear that whether a referent is located within (‘peripersonal space’) or beyond (‘extrapersonal space’) a speaker’s physical reach can influence the form a demonstrative takes in the speaker’s utterance (Caldano & Coventry, 2019; Covnety et al., 2014; Coventry, Valdés, Castillo, & Guijarro-Fuentes, 2008; Gudde, Coventry, & Engelhardt, 2016). Specifically, it has been observed for a variety of languages (Danish, English, Spanish, Ticuna) that reachable referents within an elastic zone of peripersonal physical proximity in front of the speaker typically elicit more ‘proximal’ demonstratives than referents located beyond the speaker’s reach (Caldano & Coventry, 2019; Coventry et al., 2014; Coventry et al., 2008; Rocca, Wallentin, et al., 2019; Skilton & Peeters, 2020). Based on these findings, the relative location of a referent as situated within or beyond a speaker’s reach should be considered one clear factor driving a speaker’s choice for a specific demonstrative form.

A recent study suggests, however, that such speaker-anchored coding of space may not necessarily occur in communicative contexts (Rocca, Wallentin, et al., 2019). When speakers of Danish referred to shapes placed in a horizontal grid on a table in front of them, the proportion of ‘proximal’ demonstratives they used increased when the referent was physically closer to their concurrently pointing hand. Importantly, this effect was observed only when the task was performed individually or when the speaker was joined by another speaker who performed an independent, complementary task. Critically, when the task was communicative, such that the information provided by the speaker was informative and relevant to the addressee, ‘proximal’ demonstratives were anchored not to the speaker, but to the addressee or to the speaker–addressee dyad (Rocca, Wallentin, et al., 2019). This is an important finding, as referring in naturally occurring face-to-face communication is preeminently a communicative and collaborative undertaking (Apothéloz & Pekarek Doehler, 2003; Bangerter, 2004; H. H. Clark, 1996; Peeters & Özyürek, 2016).

The generalizability of findings attributing importance to the distinction between peripersonal and extrapersonal space in driving the choice of demonstrative form may hence be specific to certain contexts (Kemmerer, 1999). This idea is in line with the corpus observation that, even for languages with a relatively simple two-term system such as English, “the traditional ‘near speaker’/‘far from speaker’ distinction fails to capture the majority of phenomena in everyday spoken English in which the forms occur where there is no relation whatsoever to any physical distance from the speaker” (Strauss, 2002, p. 151). Furthermore, in contrast with theoretical accounts stressing the parallel between perceptual (peripersonal versus extrapersonal) and linguistic (‘proximal’ versus ‘distal’) representations of space in the case of demonstratives, kinematic work indicates that speakers may also sometimes prefer a ‘distal’ demonstrative for referents located within their peripersonal space (Bonfiglioli et al., 2009). Together, these findings suggest that the relative location of a referent vis-à-vis the speaker may play a role in the choice for a specific demonstrative form, but probably only in a limited number of contexts. The more important the role of the addressee in the speech situation, the smaller the influence of speaker-anchored physical factors on the speaker’s choice of demonstrative form appears to be.

The physical location of a referent can indeed be calculated in relation to the speaker, but also relative to the addressee (Brown & Levinson, 2018; Denny, 1982; Hanks, 1990; Margetts, 2018), to the speaker–addressee dyad (Hanks, 1990; Hellwig, 2018; Jungbluth, 2003; Meira & Guirardello-Damian, 2018; Peeters & Özyürek, 2016; Weinrich, 1988), or to the relation between the speaker, addressee, or dyad and some external entity such as the sea, a river, a hill (Anderson & Keenan, 1985; Burenhult, 2008; Diessel, 1999; Dixon, 2003; Levinson, 2018), or in exceptional cultural circumstances even the palace of the local sultan (van Staden, 2018). Experimental work now indeed confirms that the perspective of the addressee (Rocca, Wallentin, et al., 2019), or the speaker–addressee dyad (Peeters, Hagoort, et al., 2015), can be taken as an anchoring point (H. H. Clark, 2020) by the speaker when selecting a demonstrative form. The idea that demonstratives may in certain languages moreover sometimes specify the referent’s relative location in relation to a geographical landmark (the sea, a hill, a river, an iconic tree) as calculated from the speaker, addressee, or dyad’s point of view is present in various typological sources (Anderson & Keenan, 1985; Diessel, 1999; Dixon, 1972), but strict experimental work has not been done. Furthermore, observational and documentary work suggests that demonstrative form may also in certain languages mark the location of the referent in terms of its degree of elevation, for instance specifying to the addressee whether it is located above or below the current speech situation (Diessel, 1999). Additionally, speakers of certain languages may encode in their demonstrative choice whether a referent is located downriver or upriver from the current perspective, or whether it is moving towards the speech situation or away from it (Burenhult, 2008; Diessel, 1999; Levinson, 2018). Quasi-experimental findings confirm these typological observations for various languages (Levinson et al., 2018). In sum, the relative location of a referent vis-à-vis entities (e.g., the addressee, the dyad, a geographical landmark) *beyond the speaker alone* seems a common variable influencing the choice of demonstrative form across languages.

It is perhaps not surprising that the relative location of a referent may influence demonstrative form, as the speaker often has to identify the location of a referent anyway when deciding to produce a pointing gesture to guide the addressee’s visual attention in a desired direction. This idea suggests that demonstrative form may vary as a function of whether the speaker includes a pointing gesture in their multimodal referential utterance or not, which is confirmed by recent observations (Bohnemeyer, 2018; Brown & Levinson, 2018; Cooperrider, 2016; Cutfield, 2018; Margetts, 2018; Meira, 2018; Stevens & Zhang, 2014; Terrill, 2018; Wilkins, 2018). Hence, it may be the case that the same factor (e.g., the relative location of the referent) simultaneously influences whether a speaker produces a pointing gesture or not, and which specific demonstrative form they will use (cf. Senft, 2004). Not surprisingly, then, in sign languages used by Deaf communities, it is pointing signs that often function as demonstratives (Morford, Shaffer, Shin, Twitchell, & Petersen, 2019), suggesting a common underlying machinery.

Another physical factor that may influence the choice for a specific demonstrative form is the *visibility* of the referent. It has been claimed that several, typologically distinct languages (e.g., Quileute, Ticuna, Ute, Warao, West Greenlandic) may have one or more demonstrative forms that would be predominantly used in reference to invisible or visually obscured entities (Anderson & Keenan, 1985; Diessel, 1999; Herrmann, 2018; Meira, 2018; Skilton, 2019). West Greenlandic, for instance, is believed to have a specific demonstrative form *inna* that is opted for when speakers of this language refer to entities that are currently out of sight (Diessel, 1999). Recent experimental work indicates that also speakers of languages with a relatively simple two-term demonstrative system may take into account the visibility of a referent when selecting a demonstrative form. It has been found that speakers of English use the ‘proximal’ form *this* significantly more often for visible than for invisible referents (Coventry et al., 2014). Conversely, under similar experimental circumstances, speakers of the Indigenous Amazonian language Ticuna are found to use their ‘distal’ demonstrative *ɟe*^*3*^*a*^*2*^ significantly more in reference to visible than invisible entities (Skilton & Peeters, 2020). Taken together, these experimental findings confirm earlier observations and strongly suggest that speakers may take into account a referent’s degree of visibility when selecting a demonstrative form. However, there is no universal cognitive tendency to conceptualize visible objects as relatively more ‘proximal’ (Skilton & Peeters, 2020).

### Psychological factors influencing a speaker’s choice of demonstrative form

In addition to the physical factors described above, psychological factors are found to influence a speaker’s choice of demonstrative form. These factors relate not to an entity’s objective relative physical location or visibility, but to *the cognitive status of the referent* in the mind of the speaker and/or the addressee as assumed by the speaker. It is well established that language users typically take into account the presumed cognitive status of a referent in the addressee’s situation model when using a referring expression in general (e.g., Chafe, 1976; Evans, Bergqvist, & San Roque, 2018; Gundel et al., 1993; Prince, 1981b) and when producing a communicative pointing gesture (Cleret de Langavant et al., 2011; Liu et al., 2019; Oosterwijk et al., 2017; Peeters et al., 2013; Winner et al., 2019). Important considerations for the speaker when selecting a demonstrative form may be whether the referent is in joint attention between speaker and addressee or not (Brown & Levinson, 2018; Burenhult, 2003; Evans et al., 2018; Herrmann, 2018; Knuchel, 2019; Küntay & Özyürek, 2006; Meira, 2018; Peeters, Azar, & Özyürek, 2014; Skarabela, Allen, & Scott-Phillips, 2013; Stevens & Zhang, 2013), whether it is considered perceptually, socially, and/or cognitively accessible to the addressee (Burenhult, 2008; Hanks, 2009; Jarbou, 2010; Piwek et al., 2008), and whether it can be considered in the psychologically construed shared space, the current interactional space, or within or outside the interlocutors’ conceptually defined ‘here-space’ (Cutfield, 2018; Enfield, 2003, 2018; Jungbluth, 2003; Levinson, 2018; Meira & Guirardello-Damian, 2018; Opalka, 1982; Peeters, Hagoort, et al., 2015).

Also, experienced emotions and attitudes towards the referent may come into play here. When the speaker experiences negative affect towards a referent, they may consider it psychologically distant (Levinson, 1983, 2018; Lyons, 1977), increasing the odds that they will use a ‘distal’ demonstrative form when referring to it. Indeed, “notions such as ‘near to the speaker’ may be interpreted not only in the literal, physical sense, but also by extension to ‘psychological proximity’” (Anderson & Keenan, 1985, p. 278). We consider these factors psychological and not referent-intrinsic, as the same referent may elicit different or even opposite attitudes in different speakers. Furthermore, if a referent is placed behind a physical barrier, even when physically close and visible, it may be considered by the interlocutors to be psychologically ‘not-here,’ influencing a speaker’s choice of demonstrative form (Enfield, 2018; Shin, Hinojosa-Cantú, Shaffer, & Morford, 2020). In sum, interlocutors keep track of whether a referent is psychologically proximal or distal to themselves, to the addressee, and/or to the conversational dyad, adjusting their choice of demonstrative form accordingly.

It should be noted that, in the study of exophoric demonstrative reference, it is more difficult to manipulate in an experimental lab setting the exact cognitive status of a referent in the mind of the addressee compared with, for instance, the manipulation of a referent’s spatial location or its visibility. As a spatial proxy of a referent’s psychological proximity within or outside interlocutors’ shared space, researchers have experimentally varied the location of the addressee vis-à-vis the speaker. This typically leads to a zone of physically shared space between speaker and addressee that is separate from a spatial zone outside the dyad (Coventry et al., 2008; Jungbluth, 2003; Peeters, Hagoort, et al., 2015; Skilton & Peeters, 2020). In addition, the presence or absence of visual joint attention between speaker and addressee on a referent has been experimentally manipulated to test whether this influences demonstrative production and comprehension (Peeters et al., 2014; Stevens & Zhang, 2013). Furthermore, speakers’ use of a particular demonstrative form when engaged in a joint activity has been offline correlated with the assumed cognitive status of a referent in the situation model of the addressee as judged by the researchers (Jarbou, 2010; Maes & de Rooij, 2007; Piwek et al., 2008; Shin et al., 2020). Overall, these different approaches all indicate that the psychological proximity of a referent in the mind of the addressee, as presumed by the speaker, modulates speakers’ choice of demonstrative form.

### Referent-intrinsic factors influencing a speaker’s choice of demonstrative form

Complementing physical and psychological factors, intrinsic properties or qualities of the referent and grammatical conventions play a role in the speaker’s selection of a demonstrative form. Clearly, nondeictic factors such as *grammatical gender* in many languages influence demonstrative form (cf. French *cette* *maison* ‘this house’ to *ce* *jardin* ‘this garden’). Moreover, *number* typically plays a role (cf. ‘this chair’ to ‘these chairs’), *case* may influence which specific form is used, and the *animacy*, *humanness*, or *biological gender* of the referent or even its current *posture* or *positional orientation* is in certain languages specified in demonstrative form (Diessel, 1999; Guirardello-Damian, 2018; Hellwig, 2018; Meira, 2003).

Recent experimental findings suggest that, more broadly, speakers may indeed take permanent or temporary qualities of the referent into account when selecting a demonstrative form. A referent’s *ownership* properties and its *familiarity* to the speaker have for instance been found to modulate the proportion of use of specific demonstrative forms (Coventry et al., 2014; see also Margetts, 2018). Furthermore, when speakers of Danish, English, and Italian were asked to select a demonstrative for a variety of singular nouns, without any further context, it was found that the demonstrative form they opted for was modulated by the *size* (small versus large referent) and the potential *harmfulness* (harmful referents: e.g., shark, bomb; harmless referents: e.g., lamb, tent) of the referent (Rocca, Tylén, & Wallentin, 2019). Although it remains to be established whether the experimental, online observation that the size, harmfulness, or potentially also the manipulability of a referent matters for demonstrative choice in Indo-European languages generalizes to situations of face-to-face communication (Rocca & Wallentin, 2020), it confirms that, in addition to physical and psychological factors, intrinsic properties of the referent may influence the speaker’s choice of demonstrative form.

## A conceptual framework of demonstrative reference

Our review of the experimental literature indicates, in line with earlier typological and observational work, that a wide range of physical, psychological, and referent-intrinsic factors may influence a speaker’s choice of demonstrative form. But does having a list of different influential factors mean that we fully understand what happens in the mind of a speaker when they include a demonstrative form in their verbal utterance when referring to a certain entity in a given context for a specific addressee? Ultimately, any comprehensive account of demonstrative reference should go beyond listing a couple of individual factors that may influence the choice for a specific demonstrative form in a particular language.

**Fig. 1:**
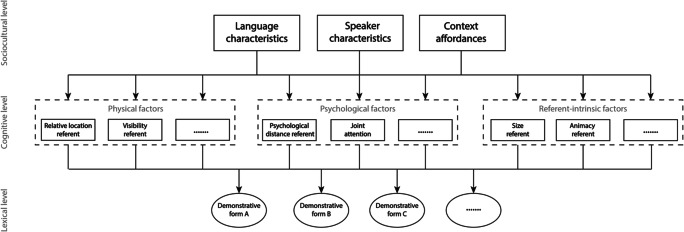
Outline of a conceptual framework of demonstrative reference, here depicted for a language with a three-term demonstrative system (depicted at the bottom, lexical level) in which several physical, psychological, and referent-intrinsic factors (nonexhaustive here, depicted at the middle, cognitive level), either categorical or continuous, influence which pronominal or adnominal demonstrative form is selected and used by a speaker. Language characteristics, speaker characteristics, and context affordances (depicted at the top, sociocultural level) in turn drive which physical, psychological, and referent-intrinsic factors are considered more important in a given sociocultural context

Figure 1 therefore provides an attempt to visually depict the minimal factors and connections that need to be in place at different levels in a conceptual framework describing demonstrative reference. The framework critically distinguishes between a lexical level (i.e., a description of the demonstrative system per se present in a specific language), a cognitive level (i.e., the range of physical, psychological, and referent-intrinsic factors that may influence the choice of demonstrative form for speakers of a given language), and a sociocultural level (i.e., how the broader cultural context, personal characteristics of the individual speaker, and the affordances of the immediate physical context shape in a top-down fashion which factors at the cognitive level play a more important role in a specific setting).

### The lexical level of the framework

The bottom, *lexical* level of the framework simply comprises the different types of demonstratives that are available to a speaker of a particular language. Languages vary substantially in the number of available demonstratives (Diessel, 1999; Levinson et al., 2018); the language-specific words, affixes, or clitics can be found in grammars of a given language. At the same time, the orthographic and phonological form, and syntactic properties of individual demonstrative terms are stored in lexical memory of proficient (and for the orthographic form: literate) speakers of the language.

As demonstratives are among the first words that we acquire in infancy (Capirci et al., 1996; E. V. Clark & Sengul, 1978), it is likely that the lexical level of the framework will be represented in a speaker’s long-term lexical memory early in life. However, adult-like, pragmatically appropriate use of these terms takes longer, potentially being fully mastered only after age 6, and possibly connected to and following the child’s development of a theory of mind (Chu & Minai, 2018; E. V. Clark & Sengul, 1978; De Cat, 2015; Gundel & Johnson, 2013; Hickmann, Schimke, & Colonna, 2015; Küntay & Özyürek, 2006; Serratrice & Allen, 2015; Tanz, 1980). The developmental gap between acquisition of the lexical items themselves and their contextually appropriate usage supports the idea that a cognitive and a sociocultural level should complement the lexical level in the conceptual framework as in the mind of the speaker.

### The cognitive level of the framework

The middle, *cognitive* level of the framework ideally comprises all factors that may influence the choice of demonstrative form in language. We have seen above that three types of factors can be distinguished: physical, psychological, and referent-intrinsic factors. We assume that many of these probabilistic factors will be continuous in nature. The relative influence of the same factor may therefore differ over time. For instance, the higher the psychological proximity of a referent to speaker and addressee becomes, all other things being equal, the higher the odds that a speaker of Dutch will select a ‘proximal’ (and not a ‘distal’) demonstrative when referring to a specific object in a given context (Peeters & Özyürek, 2016). Other factors influencing the speaker’s choice of demonstrative form may be intrinsically binary and categorical, such as whether the referent is animate or inanimate (Levinson, 2018).

Careful experimentation may disclose how physical, psychological, and referent-intrinsic properties of the referent as represented online in the mind of a speaker during a conversation may interact to lead to that speaker’s use of a particular demonstrative form in a given setting. We propose that different demonstratives may be activated at the same time in a given context in the mind of a speaker, but that only the demonstrative with the highest degree of activation will be selected and produced. Diachronic changes in the demonstrative system of a language, such as an archaic ‘medial’ demonstrative term no longer being used by speakers of a language, in the framework correspond to a gradual disappearance of the connections between all factors at the cognitive level and the specific demonstrative form at the lexical level. Furthermore, not all factors will be of equal importance in a specific language or culture, for a specific speaker, and in a specific immediate context.

### The sociocultural level of the framework

The top, *sociocultural* level of the framework therefore consists of three variables that specify in a top-down fashion which factors play a relatively more important role in the specific physical setting in which a multimodal act of demonstrative reference takes place. First, certain factors identified at the cognitive level may play an important role in influencing the choice of demonstrative form in one language, but not in another (‘language characteristics’). It has been argued, for instance, that speakers of Dyirbal take into account whether a referent is uphill or downhill from their own perspective when selecting a demonstrative form (Diessel, 1999; Dixon, 1972). It is unlikely that this physical factor would be very influential, however, in natural conversations in speakers that live in a country such as the Netherlands, where hills or other evident environmental differences in elevation are negligible.

Second, the degree to which specific factors influence demonstrative choice may differ across individuals who speak the same language (‘speaker characteristics’). If theory-of-mind development is indeed critical for the acquisition of adult-like use of demonstratives (Chu & Minai, 2018; Küntay & Özyürek, 2006), individual differences in the degree to which speakers take into account the mental state of their addressee (Apperly, 2012; Carlson & Moses, 2001) may drive whether they factor in the relation between the referent and their addressee when selecting a specific demonstrative form. Such individual differences between speakers of the same language may indeed explain part of the substantial variability observed in experiments that elicit demonstratives from different participants under virtually identical circumstances.

Studies investigating individual differences across speakers in the choice of exophoric demonstrative form are scarce. Both the broader adult literature and developmental work on the production of referring expressions, however, suggest various factors that may explain individual differences in speakers’ choice of referring expression in general (e.g., Ateş-Şen & Küntay, 2015; De Cat, 2015; Nadig & Sedivy, 2002; Serratrice & Allen, 2015; Uzundag & Küntay, 2018; Wardlow, 2013). Beyond theory-of-mind abilities (Chu & Minai, 2018; Gundel & Johnson, 2013), working memory and executive control skills may contribute to the extent speakers take into account the perspective of their communicative partner (De Cat, 2015; Nilsen & Graham, 2009; Wardlow, 2013). The amount of attentional resources available to a speaker and their capacity to inhibit and switch between perspectives may also play a role (De Cat, 2015; Long, Horton, Rohde, & Sorace, 2018). Future work is needed to test whether and how these cognitive abilities, on which individuals naturally differ, also influence a speaker’s choice of demonstrative form. We predict that individual differences in multiple aspects of executive functioning (working memory, attention, inhibition) will explain part of the variation in speaker’s choice of demonstrative form, mediated by an individual’s perspective taking and theory-of-mind skills (cf. Brown-Schmidt, 2009; De Cat, 2015).

Third, the affordances of the immediate physical and conversational context will modulate the extent to which specific cognitive factors influence a speaker’s choice of demonstrative form in a given situation (‘context affordances’). In the ‘memory game’ paradigm, for instance, the difference in physical location of the different referents is typically the most salient contextual factor that can be exploited by experimental participants in distinguishing their usage of different demonstrative forms (e.g., Coventry et al., 2008), and it is therefore not surprising that they typically do so. However, in a context in which different referents are most easily distinguishable based on, for instance, their degree of elevation, speakers may exploit that particular affordance of the given context when opting to use one demonstrative form rather than another.

## Application of the framework: The case of Spanish

To illustrate the rationale behind the conceptual framework introduced above, we will here describe how it has the potential to unite two opposite result patterns described in the literature. We will focus on the use of demonstrative determiners in Spanish, a language that has a three-term demonstrative system consisting of the basic (here singular and masculine) terms *este*, *ese*, and *aquel*. In the World Atlas of Language Structures, the Spanish demonstrative system is described as containing a three-term ‘proximal’ (*este*)–‘medial’ (*ese*)–‘distal’ (*aquel*) distance contrast (Diessel, 2013).

**Fig. 2:**
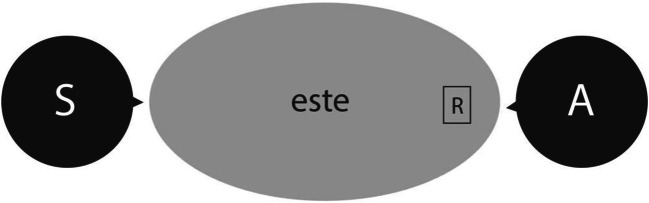
As observed by Jungbluth (2003), in naturally occurring communication, the Spanish ‘proximal’ demonstrative form *este* is dominant in reference to entities inside the face-to-face conversational dyad formed by speaker (‘S’) and addressee (‘A’). Hence, even a referent (‘R’) that is located physically close to the addressee and outside the peripersonal space of the speaker (but inside the dyad) would predominantly invite the speaker to use the ‘proximal’ demonstrative form *este* in face-to-face conversations

Jungbluth (2003), in her in-depth analysis of the Spanish demonstrative system, emphasizes that speakers and addressees when talking to each other in face-to-face situations typically “treat their shared conversational space as uniform. Everything inside the conversational dyad is treated as proximal without any further differentiation” (Jungbluth, 2003, p. 19). Crucially, she observes that in everyday Spanish conversations, the ‘proximal’ demonstrative form *este* is dominant and preferred for referents at any location inside such a face-to-face dyad, also when these are located close to the addressee and outside the speaker’s peripersonal space (see Fig. 2). This analysis is clearly not in line with traditional pure speaker-centric distance-based views of the system, which did not attribute importance to the location and orientation of the addressee in relation to the speaker in a speaker’s choice of demonstrative form (see Hottenroth, 1982). It is also not in line with a ‘person-oriented’ description of the system in which the ‘medial’ demonstrative *ese* would be predominantly used for referents that are physically located near a speaker’s addressee (Alonso, 1968).

Prima facie, the observations made by Jungbluth (2003) based on her analysis of naturally occurring interactions are conceptually difficult to reconcile with a subsequent experimental study into Spanish (and English) demonstrative use (Coventry et al., 2008). This latter study introduced the ‘memory game’ paradigm to experimentally investigate what factors influence a speaker’s choice for a specific demonstrative form. In this paradigm, participants are instructed to refer to objects that are placed at different locations on a table in front of them. In addition to the physical distance of the referent to speaker (participant) and addressee (experimenter), several theoretically interesting variables can be manipulated using the paradigm, such as the visibility of the referent object, its familiarity to the speaker, and whether it is owned by the participant or not (Gudde, Griffiths, & Coventry, 2018). Based on the theoretical account provided by Jungbluth (2003), one may predict that Spanish speakers would predominantly use *este* in reference to all entities inside the shared space between speaker and addressee when these are seated face-to-face at opposite ends of the table, regardless of the exact location of the referent on the table. After all, the table in between speaker and addressee would, at least physically, constitute the shared space between the interlocutors.

**Fig. 3:**
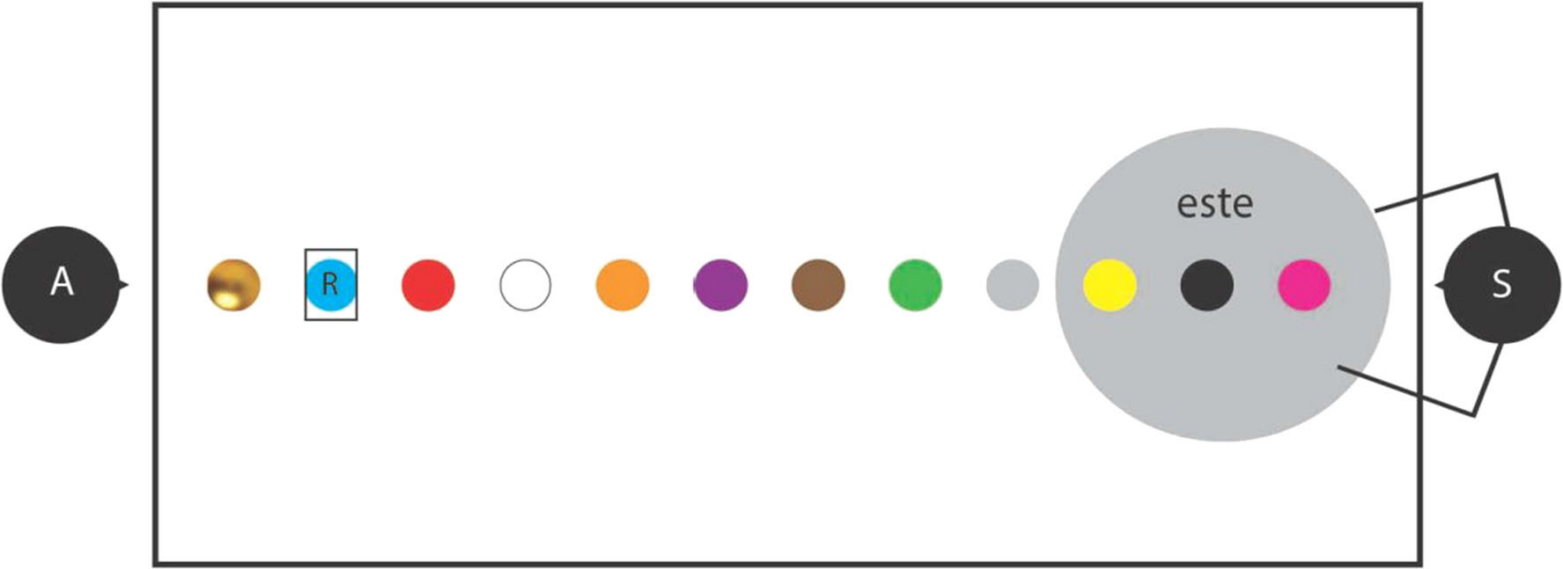
In the experimental context of the ‘memory game’ paradigm, in which a speaker (‘S’) participant and an addressee (‘A’) experimenter sit at a table, the Spanish ‘proximal’ demonstrative form *este* is dominant in reference to entities inside the peripersonal space of the speaker, as observed by Coventry et al. (2008). This spatial zone is here indicated by the large grey filled circle. A referent (‘R’) placed outside the peripersonal space of the speaker, although located inside the shared space between speaker and addressee, in this context typically does not elicit the ‘proximal’ demonstrative *este*

The study observed, however, that *este* was used dominantly only for referents inside the peripersonal space of the speaker (Coventry et al., 2008). Referents at medium distance from the speaker mostly elicited the use of *ese* and referents at a further distance from the speaker were predominantly referred to using a referential expression containing *aquel* (cf. Fig. 3). The region of space for which the ‘proximal’ form *este* was dominantly used was slightly larger when speaker and addressee were seated face-to-face compared with when they were seated side-by-side (Coventry et al., 2008), but clearly not to an extent that all referents located inside the conversational dyad were “treated as proximal without any further differentiation” (Jungbluth, 2003, p. 19). In sum, the conclusions drawn by Jungbluth (2003) based on analysis of naturally occurring Spanish interactions seem to contrast sharply with the experimental results reported by Coventry et al. (2008) on speakers of the same language. Intuitively, these results are difficult to reconcile, and one would have hoped experimental findings to generalize to naturally occurring usage patterns ‘in the wild’.

An explanation for these divergent result patterns may be found in the fact that the relative locations of the different referents, as typically indicated on the table by coloured dots (see Fig. 3) in such experimental studies using the ‘memory game’ paradigm, are highly salient to the experimental participants. The physical context hence explicitly invites speakers to exploit the relative physical location of the referent as a salient factor influencing which demonstrative form to use (cf. Enfield, 2003; Shin et al., 2020). Moreover, in the absence of a broader conversational context in which the use of the demonstratives takes place, interlocutors may have no means to jointly construe at a psychological level what they consider their shared space. In naturally occurring situations such as those observed by Jungbluth (2003), the opposite is true. Interlocutors may prefer to use demonstratives in such a way that these align with the jointly (verbally and nonverbally) construed distinction between the psychologically shared space within the conversational dyad versus any dyad-external location. In other words, speakers in the ‘memory game’ paradigm may ascribe more importance to *physical* factors such as the relative location of a referent, whereas in naturally occurring conversations *psychological* factors such as the psychological distance of a referent may play a more important role. We propose that the influence of physical factors decreases as a function of an increase of importance of the addressee in the speech situation at hand (Rocca, Wallentin, et al., 2019), and that psychological factors are by default most important in shaping a speaker’s choice of demonstrative form in natural, communicative situations.

**Fig. 4:**
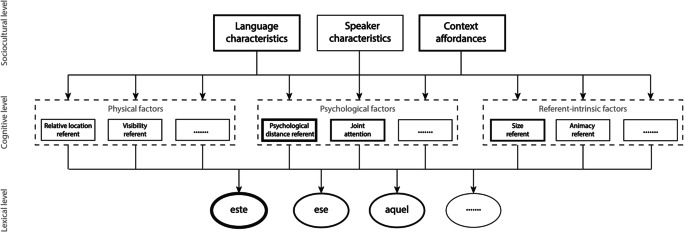
The conceptual framework of demonstrative reference, here descriptively applied to the face-to-face situation depicted in Fig. 2, inspired by Jungbluth (2003). It is assumed that in natural conversations, the psychological distance of a referent is the most important factor at the middle, cognitive level influencing the choice of a demonstrative form at the bottom, lexical level. Both language characteristics and context affordances are in a top-down fashion proposed to enhance the importance of this factor at the cognitive level in the situation depicted in Fig. 2. Because the referent is psychologically proximal, relatively small, and in joint attention, *este* is preferred over competing alternatives and therefore selected and articulated by the Spanish speaker. Based on the relative location of the referent to the speaker, both *ese* and *aquel* are arguably also considered by the speaker, but not to such an extent that they are selected for articulation

In our conceptual framework, the variable influence of physical versus psychological factors under different contextual circumstances is explained by top-down modulations of the relative importance of various factors at the middle, cognitive level as a function of the broader context affordances identified at the top, sociocultural level. Figure 4 illustrates the presumed ‘default’ situation of naturally occurring communication by speakers of Spanish. Here, we follow Jungbluth (2003) in assuming that, by definition, Spanish interlocutors aim to jointly construe a shared space and keep track of whether a referent is located inside the psychologically shared space or not (Shin et al., 2020). They adapt their choice of demonstrative form accordingly, and may even use a specific demonstrative form to indicate whether they consider a referent to be located inside the assumed shared space or not (Jungbluth, 2003; Shin et al., 2020). In line with the fact that demonstrative reference is a fundamentally social and collaborative process (e.g., Bara, 2010; H. H. Clark et al., 1983; Peeters & Özyürek, 2016), we assume that speakers implicitly consider the psychological factor ‘psychological distance of the referent’ more important than physical factors during natural conversations. Moreover, the context affordances also enhance the importance of this psychological factor as any natural face-to-face conversation allows for the construction of a shared space between interlocutors. Because the referent is located inside the shared space in the situation depicted in Fig. 2, even though it is closer to addressee than to speaker, the demonstrative *este* is strongly activated. If we here assume that the referent is relatively small in size, and that it is in joint attention between speaker and addressee, additional activation of *este* is provided through the referent-intrinsic factor ‘size of referent’ (Rocca, Tylén, et al., 2019) and the psychological factor ‘joint attention’ (e.g., Küntay & Özyürek, 2006). Because *este* is clearly more active than its competing alternatives (demonstratives *ese* and *aquel*), it will be selected for articulation by the speaker.

**Fig. 5:**
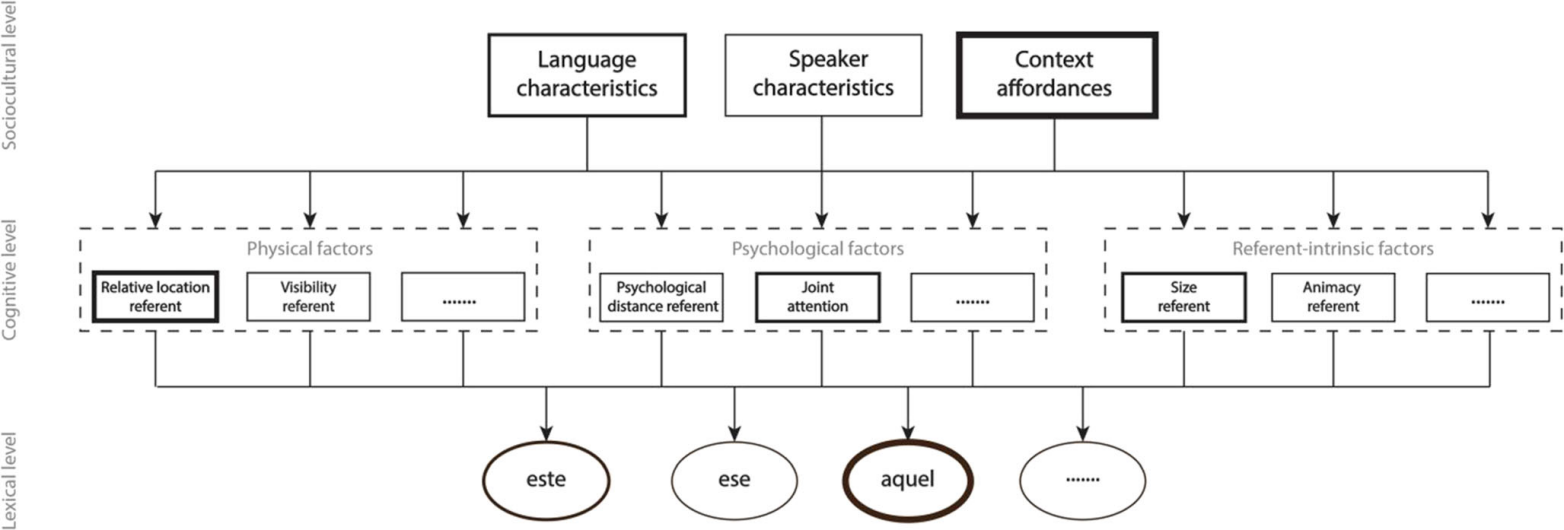
The conceptual framework of demonstrative reference, here descriptively applied to the ‘memory game’ paradigm setup as depicted in Fig. 3, and inspired by Coventry et al. (2008). It is assumed that in this experimental setup, the contextual salience (‘context affordances’) of the relative location of the referent vis-à-vis the speaker makes this latter variable the most important factor at the middle, cognitive level influencing the choice of a demonstrative form at the bottom, lexical level. Because the referent is relatively small and in joint attention, *este* is considered by the speaker. However, the top-down influence of the factor ‘relative location of the referent’ is so dominant that the referent’s relatively far location as calculated from the location of the speaker leads to *aquel* becoming activated to such an extent that it is selected for production and articulated by the Spanish speaker

The default state of the framework, in which psychological factors trump physical factors, may however be overruled, as in the context of the ‘memory game’ paradigm (see Fig. 3). In the absence of the opportunity to have a normal conversation, speakers in this context may ascribe more importance to context-dependent physical factors than to the psychological proximity of a referent in the mind of their addressee (Skilton & Peeters, 2020). The primacy of physical factors may further be primed by the salience of the different physical locations in this experimental setup (‘context affordances’) on which referents are placed. Figure 5 illustrates that for the speech situation depicted in Fig. 3, context affordances may enhance the relative importance of physical factors such as the relative location of a referent over and above the default importance of psychological factors. Because the referent is located relatively far away from the speaker in this setup, *aquel* will be activated more than *este*, explaining why it is predominantly used in reference to entities located relatively far away from the speaker.

The considerations described above may explain why in different contexts the same referent at a comparable distance from the speaker may elicit either a ‘proximal’ or a ‘distal’ demonstrative. In addition, experimental work makes clear that there are individual differences in the choice of demonstrative form across speakers of the same language under virtually identical experimental circumstances. For instance, although most participants will use a ‘distal’ demonstrative for the referent located close to the addressee in Fig. 3, some participants will use a ‘proximal’ demonstrative form in this very same context (Coventry et al., 2008). The conceptual framework explains such individual differences by assuming that factors at the middle, cognitive level of the framework may have a different default relative importance for different individual speakers. We hypothesize that individual differences in theory-of-mind capacities may contribute to whether physical or psychological factors play a more important role in different individuals. The more speakers take into account the mental states of their addressee, and as such the presumed degree of psychological proximity of a referent in the mind of the addressee, the more influential psychological factors (versus physical factors) will be in influencing a speaker’s choice of demonstrative form. Experimental research correlating speakers’ theory-of-mind capacities with their choice of demonstrative form is needed to test this proposal. Specific predictions made by our conceptual framework will be discussed more extensively in reference to Box 1 below.

## Putative parallels between exophoric and endophoric use of demonstratives

Thus far, we have focused on situations in which speakers use demonstratives exophorically (i.e., in reference to entities present in the immediate surroundings of the speech event; Halliday & Hasan, 1976; Levinson, 1983). However, in naturally occurring communication demonstratives also often function endophorically (Diessel, 1999; Himmelmann, 1996; Levinson, 1983; Lyons, 1977), when they are used in reference to elements of the ongoing spoken or written discourse. Although the exophoric use of demonstratives is considered the ontogenetic, phylogenetic, and grammatical basis from which other types of use have derived (e.g., Bühler, 1934; Diessel, 1999; Lyons, 1977; Tomasello, 2008), the endophoric use may be (even) more frequent in present-day human communication, as not only physically available referents but virtually all thinkable entities (concrete or abstract; existing or imaginary; immediately present or absent) can be linguistically introduced and endophorically referred to. Indeed, a powerful affordance of spoken, written, and signed language is that it allows one to transform any portion of discourse (e.g., a word, gesture, clause, sentence, cluster of sentences) into a newly created endophoric referent.

The main aim of this section is to explore to what extent the conceptual framework of demonstrative reference, as introduced and embedded above in an exophoric context, generalizes to situations of endophoric reference. Prior attempts to explicitly identify whether similar factors play a role in both endophoric and exophoric demonstrative use are scarce, and often restricted to the analysis of individual examples (e.g., Cornish, 1999; Fraser & Joly, 1980; Kleiber, 1983). Parallels will be explored at each level (lexical, cognitive, and sociocultural) of the framework, as well as with regard to the top-down connections between the different levels. To establish a solid basis for application of the conceptual framework to situations of endophoric demonstrative reference, we will first introduce and critically evaluate two relevant and influential existing theories of endophoric reference (the *accessibility hierarchy* and the *givenness hierarchy*), and review the experimental, qualitative, and corpus-based literature on endophoric demonstratives to disclose whether the factors that may drive a speaker’s or writer’s choice for a specific demonstrative form in a given discourse context are similar to those identified above for exophoric settings.

Before doing so, we acknowledge that different types of endophoric demonstrative use can be distinguished (cf. Cornish, 2001; Diessel, 1999; Doran & Ward, 2019; Himmelmann, 1996; Levinson, 2004). We will use the term *anaphoric* demonstrative both for demonstratives with a nominal antecedent (e.g., The Bell Jar *was first published in 1963.* *This* *is a wonderful novel.*) and for demonstratives with a propositional antecedent (e.g., The Bell Jar *was first published in 1963. This* *is something I learned in secondary school.*). This implies that we restrict the term *deictic* to nonanaphoric demonstratives in spoken and written discourse when these are used in reference to the (displaced) deictic ground (Hanks, 1992)—that is, to deictic elements of the speech or writing situation, thus covering (inter alia) situational (Himmelmann, 1996) and symbolic-exophoric (Levinson, 2004) demonstratives (e.g., nongestural deictic use of demonstratives in speech or text as in *this chapter, this year, this country, this book*). Additionally, we will distinguish between demonstrative pronouns (e.g., The Plague *was first published in 1947.* *This* *is still a highly relevant book.*) and demonstrative noun phrases (e.g., The Plague *was first published in 1947.* *This book* *is still highly relevant.*). We note that the conceptual framework likely does not generalize to situations of *cataphoric* demonstrative reference, as research in that domain shows this: strong or exclusive overall preferences for one demonstrative form (e.g., English *this*) over its alternatives (Chen, 1990; DanonBoileau, 1984; Diessel, 1999; Fraser & Joly 1980; Himmelmann, 1996; Quirk, Greenbaum, Leech, & Svartvik, 1985).

### Accessibility and givenness in relation to endophoric demonstrative form

Arguably the two most influential theories in the domain of endophoric reference are Ariel’s *accessibility hierarchy* (Ariel, 1990) and Gundel and colleagues’ *givenness hierarchy* (Gundel et al., 1993). A remarkable difference in the study of endophoric versus exophoric demonstrative use that these accounts immediately illustrate is that endophoric demonstratives have mostly been studied as part of the larger set of referring expressions available in a language, while research on exophoric demonstrative use has predominantly focused on variation within the set of demonstratives available in a language alone, as we have done above. In the former case, different types of referring expression (e.g., *the book* versus *this book* versus *it*) are argued to correspond to different cognitive statuses that a referent is presumed to have in the mental model of the reader or listener (e.g., Ariel, 1990; Gundel et al., 1993; Prince, 1981b). As such, in the study of endophoric reference demonstratives are typically seen as a small set of referring expressions within a broader range of possibilities available to the speaker or writer.

Both the *accessibility hierarchy* and the *givenness hierarchy* consistently assign demonstratives an intermediate cognitive status in between personal pronouns and definite noun phrases (Ariel, 1990; Gundel et al., 1993; Prince, 1981b). According to these views, demonstratives are used in reference to entities that are on the one hand cognitively *less* accessible than those that personal pronouns refer to, as a demonstrative (compared with a personal pronoun such as *it*) is more often found to have a nonsubject or propositional antecedent (e.g., Brown-Schmidt, Byron, & Tanenhaus, 2005; Çokal, Sturt, & Ferreira, 2018; Fossard, Garnham, & Cowles, 2012; Kaiser & Trueswell, 2008; Maes, 1997). On the other hand, demonstratives are argued to be commonly used in reference to entities that are relatively *more* accessible than those referred to by definite noun phrases (NPs). The idea is that demonstratives (e.g., “*that* *book*”) typically require a referent (e.g., “*Ulysses*”) that has been previously activated, while definite NPs (e.g., “*the* *book* Ulysses”) more commonly and more successfully introduce new referents.

The two hierarchies differ, however, as to the cognitive status attributed *within* the closed set of demonstratives. The *accessibility hierarchy* (Ariel, 1990) assumes that ‘proximal’ demonstrative forms refer to more accessible entities than ‘distal’ demonstrative forms do, and that demonstrative pronouns in general refer to entities that are more accessible than those referred to by demonstrative NPs. On the basis of distributional regularities of different demonstrative forms in a small corpus, Ariel observed that the distance between antecedent and anaphor was on average smaller for demonstrative pronouns compared with demonstrative NPs, and also for ‘proximal’ demonstrative forms compared with ‘distal’ demonstrative forms. The latter observation suggests that the simple ‘physical’ distance between antecedent and demonstrative could be an important factor driving a speaker or writer’s choice of demonstrative form. This intuitive and straightforward explanation of the difference between endophoric *this* versus *that* was, however, not confirmed by subsequent larger-scale corpus analyses (e.g., Botley & McEnery, 2001a, 2001b; Maes, 1996).

In the *givenness hierarchy* (Gundel et al., 1993), it is ‘distal’ demonstrative NPs (‘*thatN*’; e.g., ‘*that story*’) that have a special status as they are assumed to refer to entities that are currently less activated compared with entities referred to with ‘proximal’ or ‘distal’ demonstrative pronouns, or with proximal demonstrative NPs (‘*thisN*’; e.g., ‘*this story*’). This claim is arguably supported by examples of *thatN* referring to ‘familiar’ first-mention referents, reminiscent of recognitional *thatN* (Diessel, 1999; Himmelmann, 1996; Levinson, 2004; Schlegloff, 1996). Yet, one should acknowledge that familiar or recognitional *thatN* clauses are just one of many first-mention *thatN* cases, including exceptional (e.g., Chen, 1990; Cheshire, 1999; Maclaren, 1982) as well as more commonly observed first-mentions (e.g., the demonstrative form *that* or *those* followed by a noun and a relative clause: ‘*I would like to thank* *those* *people who helped us during the crisis*’). Moreover, the idea that ‘distal’ (more so than ‘proximal’) demonstratives suggest referent familiarity is challenged by analyses showing the opposite—for instance, in English evaluative discourse (Acton & Potts, 2014; Potts & Schwarz, 2010) and Swedish conversations (Lindström, 2000). Therefore, it is conceptually difficult to understand why familiar *thatN* deserves a special cognitive status compared with nonfamiliar first-mention ‘distal’ cases, or vis-à-vis other demonstrative forms. A counterexample, moreover, is indefinite *thisN,* which also represents an exceptional case of first-mention demonstrative use, but in this case of the ‘proximal’ demonstrative form *this* (Maclaren, 1982; Prince, 1981a).

In sum, both the *accessibility hierarchy* and the *givenness hierarchy* assume that differences in the presumed cognitive status of a referent in the mind of the addressee (reader or listener) are reflected by a speaker or writer’s choice of demonstrative form, but the provided evidence for these claims remains unconvincing. Of course, this does not invalidate the hierarchies as a whole, but it does question the specific assumptions they make about demonstratives. Before explaining a speaker’s or writer’s choice of endophoric demonstrative form in an alternative way in the context of our conceptual framework, we will now first review existing empirical work on the topic.

### The study of endophoric demonstrative use

In general, at least three types of methodological approaches can be distinguished in the empirical study of endophoric demonstrative reference. First, *experimental work* on the production and comprehension of demonstratives in an endophoric context is surprisingly scarce. Given the longstanding experimental tradition of investigating the cognitive status of different types of anaphors (e.g., pronouns versus nouns), it is striking that hardly any study in this domain can tell us whether there is a difference in how speakers (or writers) and listeners (or readers) produce or comprehend ‘proximal’ versus ‘distal’ anaphoric demonstrative forms. It should be relatively straightforward to carefully manipulate activation-sensitive variables like a referent’s syntactic position, its position in a sentence, or its referential distance to the antecedent in an experimental context. A notable exception (Çokal, Sturt, & Ferreira, 2014) experimentally contrasted and tested a distance-based (i.e., *that* referring to topics that were introduced earlier than *this*; cf. McCarthy, 1994) and a focus-based (i.e., *this* referring to newer information than *that*; cf. Strauss, 2002) accessibility view of the difference between ‘proximal’ and ‘distal’ demonstrative forms in an endophoric context. It is interesting that their eye-tracking and completion task results showed no straightforward correlation between the presumed accessibility of a referent and the production and comprehension of specific ‘proximal’ versus ‘distal’ demonstrative forms (Çokal et al., 2014).

Second, *qualitative studies* have provided fine-grained speculative analyses of interesting cases of demonstrative use based on acceptability judgments of either invented or naturally observed examples. Such approaches have for example identified and evaluated specific instances of recognitional *thatN* (Consten & Averintseva-Klisch, 2012), indefinite *thisN* (Maclaren, 1982; Prince, 1981a), interactional *that* (Cheshire, 1999), restrictive *that* (Maclaren, 1982), transgressive *that* (Hayward, Wooffitt, & Woods, 2015), cataphoric uses of demonstratives (Chen, 1990), emotional *that* (Chen, 1990; Lakoff, 1974), or even ‘Sarah Palin *that*’ (Acton & Potts, 2014; Liberman, 2008, 2010) and ‘Bill Clinton *that*’ (Jackson, 2013). Most of such studies focus on exceptional, often nonanaphoric or semi-anaphoric and mostly ‘distal’ cases alone rather than on the majority of demonstrative anaphors where “one could be replaced by the other with very little effect on the meaning” (Stirling & Huddleston, 2002, p. 1506). Therefore, similar to the experimental study discussed above, also qualitative studies do not convincingly disclose what factors may drive a speaker’s or writer’s choice for one demonstrative form over another in a given endophoric setting.

Third, *corpus-based studies* have been carried out with the potential to provide distributional evidence on factors influencing a speaker’s or writer’s choice of demonstrative form in endophoric use (Botley & McEnery, 2001a, 2001b; Byron & Allen, 1998; Maes, 1996; Petch-Tyson, 2000). Testing the theoretical views on demonstratives in the *accessibility hierarchy* and the *givenness hierarchy* discussed above, these studies did not offer converging evidence in favor of the presumed relation between a referent’s cognitive status and the used demonstrative form. What they firstly do show, however, is that anaphoric demonstratives (i.e., demonstratives with an NP or propositional antecedent) are in general more frequent than nonanaphoric ones. More importantly in the context of this paper, they also indicate that the relative proportions of occurrence of ‘proximal’ versus ‘distal’ demonstrative anaphors vary widely and in different directions across different corpora.

Specifically, the proportion of use of a given demonstrative form (e.g., *this* versus *that*) seems to vary strongly as a function of *text or discourse genre*. For instance, researchers in the field of English as a second language (L2) collected academic essays from students in different countries, and compared their demonstrative use with similar essays written in students’ native language (L1) (e.g., Blagoeva, 2004; Labrador, 2011; Lenko-Szymanska, 2004; Petch-Tyson, 2000; Oh, 2009). The varied results of underuse or overuse of demonstrative forms between L1 and L2 are less relevant here than the observation that on average about 70% of all demonstrative forms in all these corpora is ‘proximal’. This regularity is presumably found more generally in the broader genre of scientific, *expository* literature (Gray, 2010). Conversely, corpora of *interactional* spoken discourse consistently show (extreme) preferences for ‘distal’ anaphors (Byron & Allen, 1998; Passonneau, 1989; see also Diessel, 1999, p. 119). Such a predilection for anaphoric use of ‘distal’ demonstratives can also be found in news corpora (Botley & McEnery, 2001a) in which information is clearly targeted towards the news item’s consumer. Other genres, such as fiction or evaluative discourse, do not directly seem to result in clear preferences, probably because they represent too broad and varied text categories (Ariel, 1988; R. S. Kirsner, 1979; Labrador, 2011; Potts & Schwarz, 2010). Nevertheless, the specific text or discourse genre seems a clear and reliable top-down factor influencing a speaker’s or writer’s choice of demonstrative form (see also Gundel, Hedberg, & Zacharski, 1988).

On the basis of the experimental, qualitative, and corpus-based studies discussed above, we conclude that it is time to broaden the perspective on endophoric demonstratives by shifting attention from activation-sensitive discourse structural variables (e.g., ‘accessibility’ or ‘givenness’) to a comprehensive view that highlights the importance of the interaction between speaker (or writer), listener (or reader), and referent at a psychological level. Specifically, we propose that the bulk of anaphoric demonstratives, regardless of their specific form, expresses the same cognitive status—namely, that a referent has been or can be activated based on previous discourse information. We will argue below that the different demonstrative forms reflect subtle pragmatic and interactional inferences that significantly exceed the level of simply ‘finding the intended referent’.

### A comprehensive account of endophoric demonstrative use

The observation that text or discourse genre plays a fundamental role in driving a speaker’s or writer’s choice of demonstrative form is indeed best explained in terms of the presumed relation between speaker/writer, addressee, and referent in the mental model of the speaker/writer. We propose that an increasing preference for ‘distal’ demonstrative anaphors is observed when the role of the addressee becomes more prominent in the discourse setting at hand (as in interactional and narrative discourse), while an increasing preference for ‘proximal’ demonstrative anaphors is found when speakers feel more responsible themselves for the produced discourse, as in an expository context. Indeed, in a conversational corpus study, it was observed that “*that* frequently co-occurs with features marking interpersonal involvement in contexts where, in principle, it would seem equally possible for speakers to have chosen to use *this*. *This*, on the other hand, tends to co-occur with linguistic features that encode the speaker's own involvement in what is being said” (Cheshire, 1996, p. 375). Likewise, the strong ‘proximal’ preference shown in corpora of academic and scientific texts can be explained by an assumed primordial psychological proximity between speaker and topic in the context of an addressee to which the topic (and as such, the mentioned referents) are assumed to be psychologically more distant. At the same time, the overwhelming preference for ‘distal’ demonstratives in narrative news corpora suggests a more intensive desired interaction with and appeal to the text’s intended addressee(s). The use of a ‘proximal’ demonstrative thus locates the topic of discourse and its referents in close psychological proximity to the knowledgeable speaker or writer, while the use of a ‘distal’ demonstrative moves the referent(s) into the shared space between speaker and addressee, and as such psychologically towards the addressee.

Similar interactional inferences apply to specific types of demonstrative anaphors as well. For example, the preference in expository contexts for speakers to construe modified *thisN* anaphors may reflect that a speaker is presenting information new to the addressee (reminiscent of indefinite *thisN*). Likewise, the preference in narrative discourse for long *thatN* anaphors (reminiscent of recognitional *thatN*) suggests an appeal to the addressee to jointly engage in the narrative. Furthermore, cases of attitudinal demonstratives, predominantly ‘distal’ ones, can be seen as weak variants of (mostly) nonanaphoric pragmatic uses, with a positive appeal towards the addressee (cf. a typical greeting in Dutch such as ‘*Ha* *die* *Frits*’; literally: ‘*Hey* *that* *Frits*’, Kirsner, 1979, where the ‘proximal’ alternative is considered not a reasonable alternative).

The presumed cognitive importance of the basic speaker–addressee dyad and the relative location of a referent in their psychologically shared space is further supported by the usage patterns of typical nonanaphoric demonstratives. Deictic ‘proximal’ demonstratives, for instance, can be used as exclusive devices to refer to the nearest possible referents in the endophoric context (i.e., those in the here-and-now of discourse) and in related deictic functions, such as quoted or reported speech (e.g., in news reports, Botley & McEnery, 2001b). Furthermore, the association of ‘distal’ demonstratives with an active role of the addressee is substantiated by a larger variety of ‘loose *that*’ references, which can be read as an invitation and a signal to provide the addressee with the freedom to construct a suitable interpretation of the referent on the basis of the available contextual information. In such cases, the speaker or writer thus moves the referents psychologically towards the addressee. Indeed, ‘distal’ forms are more productive in cases of loose or deferred anaphoric reference, for example in the case of a referent shift between antecedent and anaphor (e.g. “*John’s behavior is an exact match of* *that* *of Peter*”), a shift from a specific to a generic interpretation (e.g., Bowdle & Ward, 1995), or a bridge between referents (e.g., “*A car drove by. The engine stuttered. Then another car drove by.* *That* *engine stuttered, too*”; see examples in Apothéloz & Reichler-Béguelin, 1999; Lücking, 2018).

Clearly, we do not intend to say that the role and importance of the addressee have been neglected in earlier work. On the contrary, addressee assumptions have always been crucial in defining cognitive statuses. For example, in work discussing the use of ‘familiar *that*’, the addressee is assumed to be “able to uniquely identify the intended referent because he already has a representation of it in memory” (Gundel et al., 1993, p. 278). But once we assume that most of the endophoric demonstratives easily tolerate replacement by alternative, competing demonstrative forms without ‘losing the referent’ in the mind of the listener or reader, we have to acknowledge that these purely identification-based addressee assumptions need to be updated. This conclusion is in line with the observation that “demonstrative determiners encode procedural meaning, which does not necessarily or only guide the hearer to the intended referent, but may in some cases contribute to what is implicitly communicated as well” (Scott, 2013, p. 56). In what follows, we explore how our conceptual framework of demonstrative reference incorporates this perspective on endophoric demonstratives. We will do so by distinguishing once more between the framework’s three different levels (lexical, cognitive, and sociocultural).

### The conceptual framework of demonstrative reference in endophoric settings

As to the bottom, *lexical level* of the framework, there are several languages with demonstrative forms that are exclusively used as anaphors, but in most languages the existing exophoric terms are also used in endophoric contexts (Diessel, 1999; Levinson, 2018). Therefore, the lexical level of our conceptual framework will for many languages be identical or similar across endophoric and exophoric contexts. This overlap in lexical forms used across exophoric and endophoric contexts makes it intuitively plausible that the choice of demonstrative forms in endophoric use are to a certain extent affected by the three types of cognitive variables at the middle level of the exophoric framework.

At the *cognitive level*, we previously distinguished between physical, psychological, and referent-intrinsic variables influencing a speaker’s choice of demonstrative form in exophoric settings. To what extent do these three types of factors indeed influence the use of demonstratives in reference to elements of the ongoing discourse?

First, it seems trivial that endophoric demonstratives are not sensitive to physical factors such as the visibility or relative physical/spatial location of a referent, as the endophoric referent is typically located in the ephemeral (for spoken) or displaced (for written) sphere of discourse (H. H. Clark, 2020). We have seen that the ‘physical distance’ between referent and antecedent has been proposed to drive the choice of demonstrative form (Ariel, 1990), but that this proposal was later falsified on the basis of more extensive, in-depth corpus analyses (e.g., Botley & McEnery, 2001a, 2001b; Maes, 1996). One exceptional situation in which physical factors could play a role may be found in situations where discourse topics (person, object, event) are visibly present in interactional endophoric contexts. However, it is questionable whether in such contexts the demonstrative is used purely endophorically. In sum, as in exophoric settings (Peeters & Özyürek, 2016), it is not physical factors that are primary in driving an individual’s choice of endophoric demonstrative form.

Second, psychological factors seem fundamental in driving a speaker’s or writer’s choice of endophoric demonstrative form by shaping the interaction between speaker, addressee, and referent. We assume that speakers and writers commonly keep track of the psychological proximity of a referent in their own mental model in relation to the mental model of their addressee, and the degree of assumed joint attention between speaker/writer and addressee on the referent. The chosen demonstrative form will often reflect the relative position of the speaker or writer in relation to the addressee, as a function of the broader discourse genre, and discloses where exactly referents are situated in the assumed (jointly attended) shared space between speaker/writer and addressee. This can be psychologically relatively close to the speaker, as in expository contexts, or more towards the addressee, as in interactional and narrative discourse. We thus assume that the presumed psychological distance of a referent in the mind of the addressee is an important factor in driving the speaker’s or writer’s choice of demonstrative form at the cognitive level. We propose that the relative importance of this factor is top-down influenced by genre knowledge, a factor that plays a crucial role at the sociocultural level of the framework (see below).

Third, it has been hypothesized that referent-intrinsic characteristics such as animacy, manipulability, or more fine-grained semantic characteristics of a referent may implicitly guide a writer’s choice of demonstrative form (Rocca, Tylén, et al., 2019; Rocca & Wallentin, 2020). It remains to be tested whether such subtle influences manage to beat genre affordances or interactional strategies of speakers (see below). Considering potentially large effects of text genre on endophoric demonstrative variation, the influence of referent-intrinsic factors on the choice of endophoric demonstrative form may be relatively small (Maes, Krahmer, & Peeters, 2020). Nevertheless, the current status of a referent in the presumed common ground between speaker and addressee could represent one flexible referent-specific variable influencing a speaker’s choice of demonstrative form. In a study of language use in contexts of negotiation, a systematic difference between unresolved (‘proximal’) and resolved (‘distal’) negotiation topics was observed (Glover, 2000)—a dichotomy which can easily be interpreted as reflecting a difference in spatiotemporal—and, consequently, psychological—distance between interlocutors and the referent as a function of its current status (near, current, still under discussion versus far, past, finished). As such, the communicative status of a referent could influence a speaker’s choice of endophoric demonstrative form as a temporary and flexible referent-intrinsic factor.

On the *sociocultural level*, we consider the affordances provided by genre-related knowledge as most crucial in influencing demonstrative variation in a top-down fashion. Text or discourse genre, as such, is the endophoric counterpart of the exophoric ‘context affordances’ we discussed before. In spoken interaction, these affordances themselves differ from what we discussed in the exophoric sections, as the prototypical situation of two interlocutors engaged in talking about spatially arranged (and sometimes competing) visible objects only represents one aspect of natural conversations. Instead, we consider the possibility to have a physical interaction with an addressee as the crucial predictor for the endophoric ‘distal’ preference in narrative and interactional settings, as it enables speakers to immediately express their social intention to create joint attention to a nonphysical referent with the addressee. More broadly, specific cultural genre knowledge (‘language characteristics’) can afford and stimulate a large range of assumed relations between speaker, addressee, and referent.

In addition to context affordances such as text and discourse genre, we predict that personal characteristics of the speaker or writer are crucial for their choice of demonstrative form, also in endophoric settings. Endophoric referential choices are based on speakers’ assumptions rather than on immediately observable evidence (Prince, 1981b, p. 232). Choices can differ across individuals and contexts, because discourse conditions not always allow for a univocal choice, and speakers will differ in their ability to construct adequate assumptions about the mental model of their addressee(s). This may be due to individual speaker differences in memory span and theory-of-mind abilities, or because speakers take the freedom to deviate from the referential default—for instance, by purposefully using a first-mention demonstrative or demonstrative NP rather than a simple pronoun. For activation-based expressions, speakers’ leeway is intelligently covered by the idea that cognitive statuses are *implicationally* related, predicting that “a form can appropriately encode the necessary and sufficient status (the status immediately above the form in the table) as well as all higher statuses” (Gundel et al., 1993, p. 290). But once we assume that demonstrative forms largely encode the same cognitive status, it is reasonable that they will show relatively more individual and less systematic variation than other types of referring expression. Speakers with stronger theory-of-mind abilities, relatively more genre knowledge, or enhanced general rhetorical skills will be able to exploit putative implicational differences between different demonstrative forms more extensively and more strategically than others. Furthermore, individual variation in choice of demonstrative form will vary as a function of the degree to which discourse genre characteristics have been contextually specified.

In sum, we argued in this section that different endophoric demonstratives typically access referents with the same or a similar cognitive status, and that they carry subtle pragmatic inferences related to the presumed relation between speaker, addressee, and referent at a psychological level. We assume that cognitive abilities and stylistic, rhetorical skills of individual speakers and writers lead to substantial variation in their choice of demonstrative form, and consider (cultural knowledge on) genre affordances as the most predictive top-down variable explaining the distribution of endophoric demonstratives across different contexts. This knowledge is informative about the position of the speaker or writer in relation to their addressee(s), and influences where exactly referents will be situated in the assumed (jointly cognitively attended) shared space between speaker/writer and addressee. Physical factors and referent-intrinsic variables on the cognitive level are considered less influential.

Clearly, much work remains to be done to validate or reject our conceptual framework of demonstrative reference, also with regard to its endophoric predictions. First, we need more reliable corpus evidence (natural and elicited) that directly compares the use of demonstratives across discourse genres. The development of a decent endophoric toolbox, comparable with the one in use for elicitation of demonstratives in exophoric settings (Wilkins, 2018), would be helpful in this respect. —Second, more experimental evidence is needed, for instance, through controlled experiments investigating the effect of genre on individuals’ choice of demonstrative form in different contexts, and on individual cognitive variability in relation to genre knowledge and genre specificity.

## Future directions

In this paper, based on *previous* work, we introduced a novel conceptual framework of demonstrative reference. Box 1 summarizes a set of 10 testable predictions that our conceptual framework makes, which can be investigated by *future* work on demonstratives. Box 2 additionally presents several open questions in the study of demonstrative reference. In this penultimate section, we will discuss such remaining open questions and look out on promising developments in the study of demonstrative reference and its applications.

**Box 1** Ten testable predictions derived from the conceptual framework of demonstrative reference introduced in this paper

**Table:** 

1. Physical, psychological, and referent-intrinsic factors jointly influence a speaker’s choice of exophoric demonstrative form in any given communicative setting. 2. The relative importance of these three types of factor differs as a function of the affordances of the specific speech situation. 3. In natural, communicative situations, psychological factors are by default more influential than physical factors in shaping a speaker’s choice of exophoric and endophoric demonstrative form. 4. The more important the role of the addressee in the speech situation, the smaller the influence of speaker-anchored physical factors on the speaker’s choice of demonstrative form. 5. The relative influence of physical versus psychological factors in shaping speakers’ and writers’ choice of demonstrative form varies as a function of their theory-of-mind capacities. 6. Languages differ in the relative importance of individual physical, psychological, and referent-intrinsic factors that influence a speaker’s choice of demonstrative form in a given language. 7. Discourse genre is the most important predictor of a speaker’s or writer’s choice of endophoric demonstrative form. 8. Expository discourse will elicit clear overall preferences for the use of ‘proximal’ demonstratives, whereas interactional and narrative discourse will elicit clear overall preference for ‘distal’ demonstratives. 9. The bulk of anaphoric demonstratives, regardless of their specific form, express the same cognitive status—namely, that a referent has been or can be activated on the basis of previous discourse information. 10. The conceptual framework of demonstrative reference also to a large extent explains the form and kinematics manual pointing gestures take.	

**Box 2** Outstanding questions in the study of demonstrative reference

**Table:** 

1. To what extent does the conceptual framework of demonstrative reference as depicted in Fig. 1 generalize to cases of definite and indefinite reference (e.g., noun phrases including definite and indefinite articles) beyond demonstratives? 2. Why do speakers select demonstratives (versus alternative referring expressions) in the first place? 3. To what extent do the factors at the sociocultural and cognitive level of the framework play a role in the mind of the *addressee* when *comprehending* a demonstrative? 4. What is the extent of variability across languages in terms of the basic configuration of the conceptual framework? 5. To what extent do similar factors drive the speaker’s choice of demonstrative form in contrastive and noncontrastive situations of exophoric demonstrative use? 6. To what extent can corpus data and experimental findings be used to determine the overall extent of individual variation in speakers’ choice of demonstrative form? 7. What are the basic parameter settings of a computational implementation of the conceptual framework? 8. What brain structures and networks support the online production and comprehension of demonstrative reference?	

### Beyond demonstratives: Referring expressions in general

Our review of the literatures on exophoric and endophoric demonstratives revealed an interesting difference between these two related, but often distinctly approached topics of study. We saw that endophoric demonstratives are typically considered and studied as part of a larger set of referring expressions available to the language user, whereas research on exophoric demonstratives often focuses on the various factors influencing a speaker’s choice of one demonstrative form versus another. This discrepancy in empirical scope naturally raises the open question of whether the conceptual framework of demonstrative reference, as introduced in this paper, generalizes to a broader set of referring expressions (e.g., definite and indefinite articles, personal pronouns such as English’s *it*) *beyond* demonstratives. In the case of exophoric reference, for instance, do the various physical, psychological, and referent-intrinsic factors identified at the middle, cognitive level of the framework also influence whether speakers will use a demonstrative (versus an alternative referring expression) at all? In the case of endophoric reference, for example, how influential is discourse genre in driving speakers’ choice of any referring expression on the scale between zero anaphora and full definite expressions?

A few studies in the exophoric domain have, as in the endophoric domain (Acton & Potts, 2014; Wolter, 2006), explicitly investigated why speakers use a demonstrative (versus an alternative referring expression) in the first place (Bangerter, 2004; Cooperrider, 2016). It has been proposed that an important function of a demonstrative is to focus the addressee’s attention on the concurrent, deictic pointing gesture, particularly in situations where that gesture provides unambiguous and critical information about where a referent can be found (Bangerter, 2004; Bühler, 1934; Cooperrider, 2016). As such, referents that are contextually ambiguous—for instance, because they are located relatively far away and in the presence of competitor objects—may elicit referring expressions that contain more detailed verbal information beyond a demonstrative. Successful future study of the relation between demonstratives and other referring expressions will therefore require the experimental exophoric researcher to not restrict their participants to the use of demonstratives alone (cf. Coventry et al., 2008).

A promising development in this vein is presented by a recent cross-linguistic study in which a well-established experimental paradigm to study exophoric demonstratives (in isolation) was extended to study the use of demonstratives versus definite and indefinite articles (Skilton & Peeters, 2020). This study observed that speakers of Dutch (the Netherlands) consistently preferred to use noun phrases containing a *definite article* in reference to objects that had been recently introduced and were in cognitive joint attention between speaker and addressee (cf. Coello & Bonnotte, 2013; Kirsner, 1993). Speakers of the Amazonian language isolate Ticuna (Peru), however, consistently used *demonstrative* noun phrases in reference to the same objects under similar experimental circumstances. This finding suggests that there may be interesting observations to be made once exophoric researchers start broadening their horizons towards studying referring expressions beyond demonstratives. Furthermore, it raises the question to what extent there is variability across languages in terms of the basic configuration of the conceptual framework in general, and when extended to include various referring expressions beyond demonstratives.

A related open issue is the question whether our framework generalizes both to situations of contrastive and noncontrastive demonstrative reference. In exophoric contexts, demonstratives are often implicitly or explicitly used *contrastively* (“this object, not that one”), and current experimental approaches commonly elicit demonstratives in implicitly contrastive setups (e.g., Coventry et al., 2008; Rocca, Tylén, et al., 2019). In the endophoric domain, examples of contrastive demonstrative use are rare (Maes, 1996). Future work may therefore test whether similar factors play a role in contrastive and noncontrastive situations of exophoric demonstrative reference.

### Beyond demonstratives: The form and kinematics of pointing gestures

Another open issue is the extent to which our conceptual framework may describe and explain not only a speaker’s choice of demonstrative form, but also the exact form their pointing gesture takes when they refer to something. Three observations suggest that there may be high degrees of overlap in the mechanisms involved in the speaker’s selection of a specific demonstrative form, as described by our framework, and their selection of a type of pointing gesture (e.g., index-finger pointing, thumb pointing, whole-hand pointing) and its specific kinematics (e.g., fast versus slow movement; small versus large gesture).

First, it has been widely observed cross-linguistically that the demonstrative forms speakers predominantly use differ for referents located in the space directly in front of them compared with referents located behind them (Levinson, 2018). This distinction seems to align well with the fact that in many language communities, speakers often point with their thumb when a referent is located behind them, and with their index-finger when a referent is located in front of them (e.g., Kendon & Versante, 2003). Furthermore, referents in a relatively more distant location typically elicit pointing gestures that have a larger stroke amplitude compared with referents that are located relatively more nearby (Gonseth, Kawakami, Ichino, & Tomonaga, 2017; Gonseth, Vilain, & Vilain, 2013). Thus, the *relative location* of a referent may influence the form a pointing gestures takes, in terms of both its type (e.g., index-finger versus thumb) and the specific kinematic parameters (e.g., stroke amplitude) of the token.

Second, it has been observed that invisible referents, such as when giving an addressee directions in the streets towards a currently invisible end point, often elicit whole-hand pointing gestures, whereas visible referents may be more typically referred to using index-finger pointing (Flack, Naylor, & Leavens, 2018; Wilkins, 2003). Likewise, congenitally blind speakers, as well as blindfolded speakers, are observed to primarily use whole-hand gestures (rather than index-finger pointing gestures) when pointing at (invisible) objects (Iverson & Goldin-Meadow, 2001). These observations seem to align with the fact that *visibility* may impact speakers’ choice of demonstrative form, as incorporated in the conceptual framework of demonstrative reference.

Third, experimental studies have observed that speakers meticulously tailor the kinematics of their index-finger pointing gestures to the communicative needs of their addressees (e.g., Cleret de Langavant et al., 2011; Liu et al., 2019; Peeters, Chu, et al., 2015). For instance, speakers commonly lower the velocity of their pointing gesture, and keep their index finger in apex position for a significantly longer time interval, when a referent is assumed to be communicatively more relevant to the addressee (Peeters et al., 2013). Arguably, this offers the addressee more time to correctly detect the location and identity of the intended referent. These experimental findings are in line with the observation that pointing gestures in natural interactions differ in size as a function of whether they carry more or less foregrounded information for the addressee (Enfield, Kita, & de Ruiter, 2007). As such, these observations also nicely align with the finding that speaker’s choice of demonstrative form varies as a function of the presumed *communicative relevance* of a referent for the addressee (Rocca, Tylén, et al., 2019).

Taken together, it seems that similar factors (e.g., the relative location of a referent, its visibility, and its presumed cognitive status in the mind of the addressee) shape a speaker’s choice of demonstrative form as well as the form and kinematics of their pointing gesture. Similar top-down factors (language characteristics, speaker characteristics, and context affordances) may furthermore influence which of these cognitive factors play a more important role in shaping the form and kinematics of a pointing gesture in a given context (Cooperrider, 2020; Kita, 2003). Language communities differ (‘language characteristics’) in the overall proportion of use of specific articulators (hand, nose, chin, etc.) when pointing (Cooperrider & Núñez, 2012; Cooperrider, Slotta, & Núñez, 2018; Enfield, 2001; Orie, 2009; Sherzer, 1973). Individuals will differ (‘speaker characteristics’) in the form their pointing gesture will take under similar circumstances, as the relation between pointing and individual differences in theory-of-mind development has been clearly established (e.g., Baron-Cohen, 1989; Camaioni, Perucchini, Bellagamba, & Colonnesi, 2004; Tomasello, Carpenter, & Liszkowski, 2007). The broader physical and social context may again modulate which cognitive factors are considered more important in a given setting (‘context affordances’).

In sum, we thus propose that our conceptual framework of demonstrative reference may generalize surprisingly well to manual ways of referring. Both for speech communities that use the hands in various ways to point, and for speech communities that commonly point using articulators beyond the hands (e.g., the chin, nose, lips) in addition to manual articulators, the same factors that influence a speaker’s choice of demonstrative form may also influence the form and kinematics of their pointing gestures. More work is needed to specifically test these proposed parallels in the mechanisms leading to the articulation of demonstratives and gestures.

### Demonstrative reference during development

In the Introduction, we described demonstrative reference as a joint action, in which both speaker and addressee have a pivotal part to play. The importance of the speaker–addressee *dyad* and their interaction in the process of establishing joint attention on a referent becomes immediately evident when looking at situations of demonstrative reference during (ontogenetic) development. In prototypical triadic situations, infant and caregiver may actively use eye gaze, gesture, facial expressions, and spoken words such as demonstratives to share attention to a referent and *jointly* establish the topic of their ongoing interaction (e.g., Bakeman & Adamson, 1984; Mundy & Newell, 2007; Rodrigo, González, de Vega, Muñetón-Ayala, & Rodríguez, 2004; Tomasello et al., 2007; Yu & Smith, 2017). Indeed, the infant literature on the acquisition of reference confirms that language (including its nonverbal aspects) should be seen as “the vehicle for the exchange of a message that requires both a speaker and an addressee” (Serratrice & Allen, 2015, p. 6). A key question in this domain is what the exact trajectory is of the development of the cognitive underpinnings that underlie the human capacity to refer (e.g., De Cat, 2015; Hughes & Allen, 2015; Küntay & Özyürek, 2006; Serratrice & Allen, 2015).

The relatively small number of studies that have explicitly focused on the acquisition of demonstratives make clear that demonstratives as lexical items are acquired early during development, but that it may take years for the child to reach an adult-like use of these terms (Capirci et al., 1996; Chu & Minai, 2018; E. V Clark & Sengul, 1978; Küntay & Özyürek, 2006; Rodrigo et al., 2004; Tanz, 1980; Wales, 1986). Considering our conceptual framework, these observations suggest that the lexical level of the framework is acquired first, whereas more time is required to develop the relevant cognitive skills and sociocultural knowledge to use demonstratives in a pragmatically appropriate way. This idea is confirmed by the infant literature on the acquisition of reference more broadly. Also when looking at referring expressions beyond demonstratives, the acquisition of the referential lexical items typically precedes their adult-like use, which itself is dependent on, for instance, the child’s development of executive control and theory-of mind skills (Ateş-Şen & Küntay, 2015; De Cat, 2015; Gundel et al., 2013; Nadig & Sedivy, 2002; Serratrice & Allen, 2015; Uzundag & Küntay, 2018). An interesting avenue for future research would be to study how such speaker characteristics interact with language characteristics and context affordances during different stages of development with regards to the use of demonstratives and referring expressions more broadly (cf. Chu & Minai, 2018).

### Demonstrative reference in human–computer interaction

Thus far, we have approached the use of demonstratives from a theoretical point of view. The study of demonstrative reference, however, also has relevant practical implications. Ever since researchers have started thinking about natural, spoken interactions with computer systems—and long before such systems became a real possibility, as they are now, with virtual assistants like Siri, Cortana, and Google Assistant—the possibility of using deictic gestures to point the computer’s attention to an object has been explored. One of the best-known examples in this vein is arguably described by Bolt (1980), who proposed to combine speech and gesture as a new, natural input modality in a graphical user interface. Using the (at the time) nascent technologies of speech recognition and location sensing, Bolt’s system could automatically interpret an exophoric instruction like ‘put that there’, where ‘that’ was understood to refer to ‘whatever is pointed at’ (Bolt, 1980). Systems in this mould often model physical properties of the target referent, such as its size and physical distance, as formalized in Fitts’s law (Fitts, 1954; MacKenzie, 1992), explaining why targets that are closer and larger are relatively easier to point at compared with targets that are smaller or further away. Generally, the spoken utterance accompanying the pointing gesture has received little attention in those endeavours. But exceptions exist, like van der Sluis and Krahmer (2007), who focus on the trade-off between information in gesture and in words, predicting that imprecise pointing gestures are more often accompanied with more extensive verbal information, while more precise pointing is accompanied with less verbal information. Importantly, however, in none of these approaches is any attention devoted yet to the choice between ‘this’ versus ‘that’. Future work could incorporate theoretical insights on demonstrative reference into systems that allow for human–computer interaction.

## Conclusion

This paper introduced a conceptual framework of demonstrative reference. Based on a review of the literature, we proposed that physical, psychological, and referent-intrinsic factors dynamically interact to influence what demonstrative form a speaker will use in a given context. However, the relative influence of these factors themselves was argued to be a function of the cultural language setting at hand, the theory-of-mind capacities of the speaker, and the affordances of the specific context in which a speech event takes place. We showed that the framework is capable of reconciling seemingly irreconcilable results, and that it may to a large extent generalize to situations of endophoric reference and to the production of pointing gestures.

Two natural next steps are to formalize and computationally implement the current framework, and to further identify the neural architecture supporting demonstrative reference. Existing computational models of language production may shed further light on when and why a speaker would use one demonstrative form or another. The framework we have proposed includes a variety of influential factors, and computational models precisely force one to be fully explicit about the model factors and their contributions, thereby also potentially furthering our understanding of the interplay between them (e.g., Frank & Goodman, 2012; Goodman & Frank, 2016; Lewandowsky & Farrell, 2010; van Deemter, Gatt, van Gompel, & Krahmer, 2012; van Gompel, van Deemter, Gatt, Snoeren, & Krahmer, 2019). At a neurobiological level, a first handful of studies suggest that demonstrative reference is supported by an interplay between the perisylvian language network, the theory-of-mind network, and a visuo-attentional network, together supervised online by activation of areas involved in cognitive control (Brunetti et al., 2014; Cleret de Langavant et al., 2011; Committeri et al., 2015; Peeters, Snijders, Hagoort, & Özyürek, 2017; Rocca, Coventry, et al., 2019). Future work in this domain may link such networks to aspects of our framework.

To conclude, what this paper as a whole makes clear is that reaching a full understanding of demonstrative reference requires combining insights from various academic disciplines. Close collaboration is needed between (i) linguists-anthropologists typologically describing the demonstrative systems of the different languages of the world and identifying factors that might influence the choice of demonstrative form in a particular language on the basis of in-depth documentary and corpus-based work; (ii) experimental psychologists testing for the unique contribution of a proposed factor in different languages and different experimental contexts and testing to what extent certain factors influencing the choice of demonstrative form are universal or language-specific; (iii) computational linguists incorporating demonstrative reference in computational models of language production to specify the mechanisms involved in the speaker’s choice of demonstrative form, leading to new hypotheses for experimental psychologists to empirically test; and ultimately (iv) neuroscientists specifying the underlying neural infrastructure and its dynamic activation in supporting the online selection of demonstratives in naturally occurring multimodal communication. Demonstratives should best be studied in the context of pointing gestures and both from an exophoric and endophoric perspective in relation to other referring expressions, in both infants and adults. We believe this multidisciplinary endeavour is worth undertaking, as the fundamental importance of demonstrative reference for human communication cannot easily be overstated.
